# Acute ischemic stroke after enjoying Top of Europe

**DOI:** 10.1002/ccr3.4016

**Published:** 2021-05-06

**Authors:** Marina P. Krasteva, Katarzyna Pospieszny, Mirjam R. Heldner

**Affiliations:** ^1^ Department of Neurology Inselspital University Hospital and University of Bern Bern Switzerland; ^2^ Institute of Diagnostic and Interventional Neuroradiology Inselspital University Hospital and University of Bern Bern Switzerland

**Keywords:** all cerebrovascular disease/stroke, MR imaging, stroke etiology

## Abstract

Sudden onset of disturbed consciousness, neurocognitive deficits, and weakness of the proximal limbs are typical findings of a watershed stroke. Occurrence after an intense emotional experience and electrocardiogram changes are hints toward the rare cause of stroke of a takotsubo cardiomyopathy, even more if the stroke pattern is embolic.

## Graphical abstract

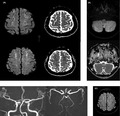

Takotsubo cardiomyopathy is a temporary heart condition. It typically occurs after an intense—usually negative—emotional experience or physical stress and thus is also known as stress cardiomyopathy or broken heart syndrome. Linked to impaired blood pump activity and/or arrhythmia, ischemic strokes of mostly embolic pattern have been described.

A 58‐year‐old so‐far healthy female Asian tourist was reportedly positively overwhelmed by the breathtaking view at the Jungfraujoch (3454 m.a.s.l.). While descending with the railway, she suddenly experienced chest pain and shortness of breath. She went to the toilet, where, within minutes, she was detected lying on the floor, verbally responsive and cursorily oriented but somnolent and showing tetraplegia. The paramedics arriving at the scene suspected an acute stroke and flew her to our stroke center by air ambulance. On emergency admission, examination revealed a residual moderate right‐sided proximal paresis and neglect as well as somnolence and apathy. Cerebral magnetic resonance imaging showed findings (Figure 1), indicating former hemodynamic compromise and explaining the clinical neurological findings. Cardiac investigations revealed a pathological electrocardiogram with negative T waves in the area of the anterior wall (Figure 2), elevated cardiac biomarkers, large apical akinesia with basal hypercontractility in transthoracic echocardiography, and a normal coronary angiography, being consistent with takotsubo cardiomyopathy^1^, which was triggered by intense positive emotions causing a rare stroke etiology. Takotsubo cardiomyopathy is a temporary heart condition. It typically occurs after an intense—usually negative—emotional experience or physical stress and thus is also known as stress cardiomyopathy or broken heart syndrome. Linked to impaired blood pump activity and/or arrhythmia, ischemic strokes of mostly embolic pattern have been described.^1^, ^2^ What is special about our reported patient is that the ​takotsubo cardiomyopathy occurred after an intense positive not negative emotion and caused an ischemic stroke of a hemodynamic pattern.

**FIGURE 1:**
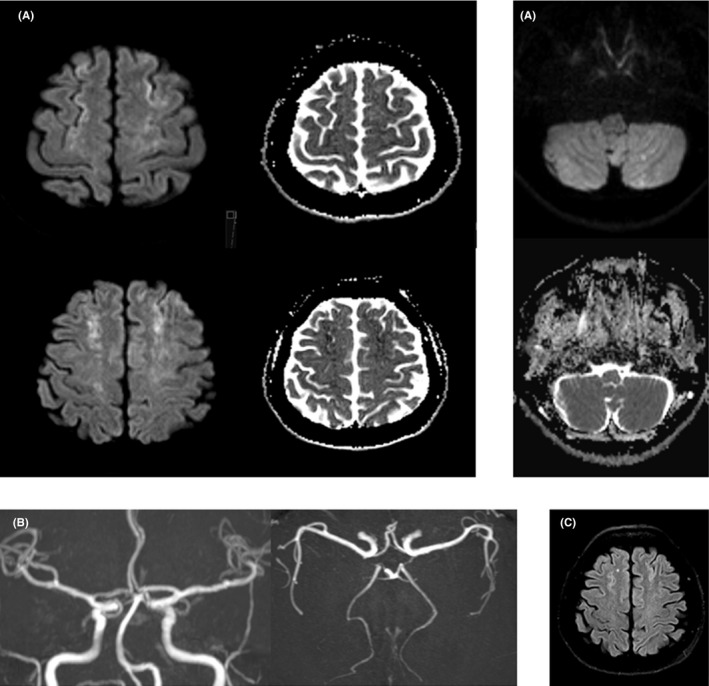
A, Diffusion‐weighted imaging (b1000) and apparent diffusion coefficient (ADC) map: Cortical restricted diffusion along the watershed zones (middle cerebral and anterior cerebral artery/posterior inferior cerebellar artery and superior cerebellar artery) with correlation on the ADC‐map as a sign of ischemia. B, Time‐of‐flight angiography: no signs of vasoconstriction or thrombus. C, Fluid‐attenuated inversion recovery (FLAIR) imaging: cortical ischemic demarcation along the bifrontal watershed zones

**FIGURE 2:**
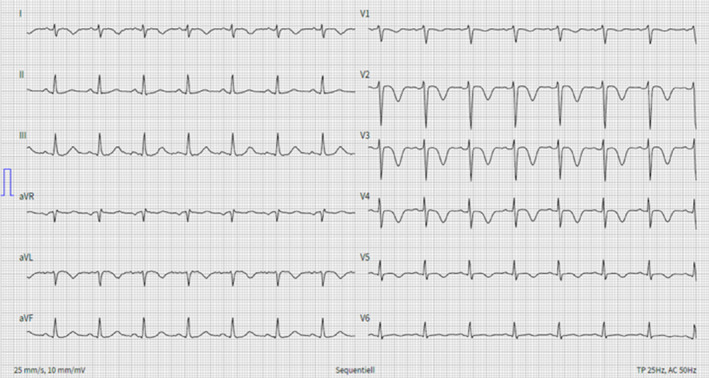
Electrocardiogram: negative T waves in the area of the anterior wall

## 1 CONSENT

Written informed consent was obtained from the patient for the publication of this clinical image.

## ETHICS APPROVAL AND DATA AVAILABILITY STATEMENT

For publication of case reports, ethical approval is not needed by our local ethics committee. Data included in this case report can be made available upon request to the corresponding author.

## CONFLICT OF INTEREST

The authors have no conflicts of interest to declare.

## AUTHOR CONTRIBUTIONS

Marina P Krasteva, MD, Department of Neurology, Inselspital, University Hospital and University of Bern, Switzerland: acquired, extracted, and interpreted the data, drafted the manuscript, critically revised the manuscript for important intellectual content, and gave final approval for publication. Katarzyna Pospieszny, MD, Institute of Diagnostic and Interventional Neuroradiology, University Hospital and University of Bern, Switzerland: acquired and interpreted the data, drafted the manuscript, critically revised the manuscript for important intellectual content, and gave final approval for publication. Mirjam R Heldner, MD, MSc, Department of Neurology, Inselspital, University Hospital and University of Bern, Switzerland: supervised the study, acquired and interpreted the data, critically revised the manuscript for important intellectual content, and gave final approval for publication.
